# Gynecological and Obstetrical Society of Baltimore

**Published:** 1895-06

**Authors:** William S. Gardner

**Affiliations:** Secretary; 613 Park Ave


### REGULAR MEETING OF GYNECOLOGICAL AND OBSTETRICAL SOCIETY OF BALTIMORE.

The President, Dr John Neff, in the Chair.

Dr. Witner Brinton reported "A Recent Craniotomy."

In the practice of obstetrics in the past, when a difficult case of labor occurred, due to the influence of a moderately contracted pelvis, or a very large child, the obstetrician delivered the child either with high forceps or podalic version, choosing one of these measures, which by education or predilection he most favored. All of us can remember the heated discussions in the past between the partisans of these two methods.

In the more difficult cases, where the pelvis is so contracted that a living child cannot be delivered at full term by either forceps or version, symphyseotomy or Cæsarean section are the measures to be adopted with the exception of an occasional case of induced premature labor, which may give a puny youngster the chance of being later numbered among those who are fit to live in the battle of life.

Each of these operative measures has a distinct field, but it is not my purpose to-night to speak of the special indications of each measure; I only wish to refer to the operative meas ure termed craniotomy. This term is applied to the perforation of the skull and the evacuation of the brain contents and is generally resorted to in cases of contraction of the pelvis' where the antero posterior diameter is 1½ and 2¾ inches and the mother refuses Cæsarean section. As the operation is performed solely in the interest of the mother, it has a wider range of applicability when the child is dead than when still living. If the child is alive, the question of destroying a living being is one of the most serious that falls to the lot of the conscientious physician.

While I am not a member, I respect the teachings of the Roman Catholic church, whose edicts against criminal abortion and craniotomy and the indiscriminate removal of ovaries should command the respect, as it has won the admiration of every thinking and well-disposed man.

Still, as far as I am concerned individually on the question of craniotomy, I do not hesitate to put myself on the side with those who will agree with Dr. Lusk when he says, "If the life of the mother is at stake, and the sacrifice of the child is necessary to her preservation, few at the present day would dispute the superiority of the mother's claim to existence."

I do not underrate the value of what might be termed the conservative measures when compared to craniotomy, viz.: symphyseotomy and Cæsarean section, which hold out the hope of saving both lives. While we hear from time to time of the successful cases of both of these operative measures by men who are specially trained in abdominal surgery, and are surrounded by the facilities and assistance pertaining to large hospitals, with their cases often selected and under their care for weeks previous to operation, I say while we hear of the successful cases, I regret that the unsuccessful cases are, as a rule, not reported, and in my opinion, if all cases operated upon, either by symphyseotomy or the Cæsarean section were placed upon record, it would show, in spite of increased knowledge, antiseptic precautions, etc., a frightful mortality to mothers and children.

The case of craniotomy which I wish to put on record to-night occurred in this city, July 18th, 1894, and was in the the practice of Dr. E. A. Smith, to whom I am indebted for the following facts in the case previous to my seeing the patient: Mrs. J. A. H——, 43 years of age, eleventh pregnancy. Had had very tedious labors in her previous confinements. Although her general health was good, locomotion had been much interfered with of recent years, on account of rapid and great accumulation of fat. When first seen by Dr. Smith, June 14th, 1894, to be engaged to attend her in July, he found her complaining of great difficulty in getting around, due to marked œdema of the lower extremities and shortness of breath; treatment was given and rest advised. Monday, July 16th, the physician was sent for, and a vaginal examination found labor beginning, and at this time the opinion was that the child's face was presenting, but as the presenting part was so high up and the woman's abdomen was so fat, this was not entirely verified. She was seen by Dr. Smith twice on Monday and three times on the following day, Tuesday. Pains had continued more or less during this time, cervix dilating slowly. The doctor was called again at 1 A. M. Wednesday, July 18th.

He found the patient having severe pains, but had not made as much progress as he expected. He remained the balance of the night and between six and seven o'clock in the morning, finding the cervix fairly well dilated, the bag of water was broken. The pains continuing and no progress being made at nine o'clock, Dr. Frey, a physician in the vicinity, was requested to see the case and to assist in the subsequent management of the same. After consultation, chloroform was given and efforts were made to deliver by means of an axis traction forceps, also with Simpson forceps. Their efforts were continued off and on for the next two hours, but no progress was made. I was requested to see the case and joined the two gentlemen between 11 and 12 o'clock. At this time the patient had been having true labor pains for fully 48 hours. I found her with a rapid pulse and an anxious face. We all agreed by this time that the child was dead. No fœtal heart could be heard after repeated examinations and the mother stated that she had not felt the movements of the child for hours.

Upon vaginal examinations at this time, I found the parts hot and dry, with a small recto-vaginal fistula near the vagina outlet, which was due to the efforts made to deliver with forceps. The presenting part was high up, the vertex presenting with the occiput to the mother's right and rear or occipito dextra posterior position. A segment of the head was in the pelvis and at this time seemed to be wedged tightly. The patient was given some stimulants and at once placed under chloroform. I applied without much difficulty Lusk's modification of Tarnia's axis traction forceps. I failed to deliver or to make the least progress in spite of great and repeated traction. Later on this effort was repeated with other forceps but with the same result. We decided, after consultation, to perform craniotomy. Not having my craniotomy instruments with me, there was a delay of nearly two hours before I saw the patient again. During this time stimulants and nourishments had been given the patient. The labor pains had returned in full force, but in spite of this a vaginal examination told us that there had been no progress. Chloroform was again given, forceps once more applied and failed to deliver. I then opened the child's cranium with a trephine perforator, washed out the contents of the same with a Davidson syringe .

I then applied Carl Braun's cranicolast and tried to extract the head, but in spite of all my efforts to do so, I failed. The greater portion of the parietal bones were removed, but the base of the child's skull would not pass through the superior strait. I finally performed podalic version and delivered the child after great difficulty. I immediately delivered the placenta by introducing my hand into the uterus. The uterus contracted finely, and much to my surprise the amount of blood lost was exceedingly small. An examination now made revealed a tear in the vagina in the posterior cul-de-sac at the uterus vaginal juncture, through which I could pass two fingers and feel the intestines. Within a short time the patient came from under the influence of chloroform and almost immediately had two or three severe vomiting spells, and was very much shocked; pulse 140 to 150; extremities. cold. Whisky was given hypodermically and by the mouth. She was made clean and comfortable. Her pulse grew stronger and she conversed with us in an intelligible manner, expressing great satisfaction that she had passed through the severe ordeal and that all was over. I left the patient at 4 P. M. and did not see her again. I am indebted to Dr. Smith for the subsequent history of the case. Under date of October 12, 1894, he writes as follows:

"*Dear Dr. Brinton:*—Our patient, Mrs. H., died Thursday, July 19th, about 7 P. M., being about 27 hours after her delivery. She never rallied thoroughly from the shock, her pulse ranging from 156 to 180. Although stimulants were given almost continuously, her temperature did not go much above normal until a few hours previous to her death. Shortly before dying she complained of great pain all over her abdomen, which became decidedly tympanitic. The patient finally became delirious.

"I weighed the baby on a pair of first-class meat scales; it weighed 13 pounds and 4 ounces, without the brains and parietal bones. Very high authorities claim that the brain of a newborn babe will weigh from $\frac{1}{7}$ to $\frac{1}{8}$ the weight of the whole body; so I feel safe in saying that with the loss of blood, bones, brain, etc., combined, that we had to do with a fifteen-pound babe. The gross measurements of the child were: 23½ inches long, 8 inches across the shoulders, 7 inches across the chest. I am Very respectfully, "E. A. SMITH."

I think the result of the weighing and measuring gives us the cause of the difficult labor case. A child weighing fifteen pounds, more or less, is twice the size of the average child. We had persistent occiput posterior position, with labor occurring in a woman who gave the history of always having a tedious and difficult time in her previous confinements. She was advancing in life and in a bad condition generally. I have never seen belly walls and legs so œdematous as hers were. I have performed five other craniotomies. My patients have all recovered except one who was moribund when I saw her, and I delivered her of a hydrocephalic child before death, at the request of her physicians, the late Dr. Houck and Dr. A. C. Pole. I believe it is profitable for us to write and think about our unfortunate cases. We are all anxious to let our brother practitioners, and incidentally the world, know of our successes, but as a rule we do not talk much about our failures. In this unfortunate case which I have just related, the question has come to me often, and I ask it of you to-night, what better could I have done under the circumstances related than I did? Would symphyseotomy have given me better results? I do not think so when we take into consideration the condition of the patient when I first saw her.

DR. THOMAS A. ASHBY: I have never done a craniotomy, and hope never to be forced to do one; but in the case related I do not see what better could have been done. Symphyseotomy would certainly have not given better results than were obtained. The cause of the patient's death evidently was peritonitis.

DR. B. B. BROWNE: Under the circumstances, I think the operation done was the proper one. If this child had been living, I think a symphyseotomy would have been better.

DR. J. EDWIN MICHAEL: I do not think any fault can be found with Dr. Brinton's management of this case. In July I saw a patient 5 feet, 4 inches tall, and weighing 200 pounds, whom two physicians had failed to deliver after craniotomy. I had the same trouble in delivery that Dr. Brinton had. I used Tarnier's basiotube, but failed to deliver; so I concluded to try internal podalic version, which, under the circumstances, was a very difficult operation. I succeeded in getting the legs down, but I used all my strength and weight to get the body delivered and then had much difficulty in getting the shoulders free. The child, minus the brain and skull, weighed 13½ pounds. The perineum was lacerated, but patient recovered.

The source of danger in these cases of difficulty in delivery is in the delay. If there is difficulty, we should make up our minds what should be done and then do it at once. It is evident that the longer a woman is left after operative measures have been begun, the greater the danger.

I think that craniotomy on a living child is rarely, under the present circumstances, justifiable. Symphyseotomy has filled a blank in obstetrical practice. I do not believe that a large number of unsuccessful cases of Cæsarean section and symphyseotomy have not been reported.

I believe if I had seen this patient before the death of the child, I should have performed a symphyseotomy. I think it is a good operation.

DR. WILLIAM E. MOSELY: If I had seen this patient while the child was living and the surroundings good, I would have preferred a symphyseotomy. But under the circumstances, I think the course taken by Dr. Brinton was correct.

DR. L. E. NEALE: The great trouble was in the handling of this case before Dr. Brinton was called. I think I should have resorted to craniotomy at once upon my arrival, in case I found the child dead, and I question the necessity, under the circumstances Dr. Brinton found the case, of making two more separate attempts with the forceps before resorting to craniotomy.

Whilst I advocate the importance of pelvimetry in obstetric practice, I must admit my inability to do more than vaguely approximate the size of the unborn fœtal head or to determine its adaptability to the pelvic canal.

As a matter of fact, there is a considerable difference in the size, shape, hardness, adaptability (moulding), etc. of fœtal heads at the same period of pregnancy not to mention differences in maternal pelvic structures, including both the hard and soft parts, all of which must invalidate the accuracy of pelvimetry, especially in parent.

In two cases recently confined in the Maternite, both pelves measuring the same, one was delivered by craniotomy, and the other spontaneously, without the slightest difficulty.

WILLIAM S. GARDNER, Secretary,
613 Park Ave.

